# Prevalence and association testing of antinuclear antibodies and inflammatory bowel disease in Taiwan

**DOI:** 10.7717/peerj.20474

**Published:** 2025-11-28

**Authors:** Tsai-Min Yang, Fang-Ting Lu, Hsu-Heng Yen, Yang-Yuan Chen

**Affiliations:** 1 Division of Gastroenterology, Changhua Christian Hospital, Changhua, Taiwan; 2 Frontier Molecular Medical Research Center in Children, Changhua Christian Children Hospital, Changhua, Taiwan; 3 Department of Pediatrics, Changhua Christian Children Hospital, Changhua, Taiwan; 4 Post-Baccalaureate Medicine, College of Medicine, National Chung Hsing University, Taichung, Taichung, Taiwan

**Keywords:** Inflammatory bowel disease, Crohn’s disease, Ulcerative colitis, Autoimmune, Anti-nuclear antibody

## Abstract

**Background:**

Inflammatory bowel disease (IBD), including Crohn’s disease (CD) and ulcerative colitis (UC), are characterized by chronic inflammation of the gastrointestinal tract. Antinuclear antibodies (ANAs), which are autoantibodies directed against nuclear components, are commonly present in various autoimmune disorders. We investigated the prevalence and clinical significance of ANAs in Taiwanese patients with IBD.

**Methods:**

From January 2017 to December 2024, ANA status was checked at initial diagnosis of IBD in patients from a medical center in central Taiwan. Risk factors for ANA positivity were evaluated.

**Results:**

Of the 166 patients in this study, 57 had CD and 109 had UC. ANA test results were positive (titers of ≥ 1:160) in 26 patients (15.7%). Older age at disease diagnosis (*p* < 0.05) and a diagnosis of UC (*p* < 0.05) were statistically significant risk factors for ANA positivity. Gender (*p* = 0.31), use of advanced therapy (*p* = 0.66), and the presence of extraintestinal manifestations (EIMs) (*p* = 0.14) were not associated with ANA positivity. The response to anti-tumor necrosis factor therapy did not differ between ANA-positive and ANA-negative patients (*p* = 0.34). The most frequent ANA staining patterns were AC1, AC3, and AC4.

**Conclusions:**

These findings suggest that although ANA positivity is relatively common among Taiwanese patients with IBD, particularly among older UC patients. Further validation is required to explore the clinical implications of ANA positivity in Asian population.

## Introduction

Inflammatory bowel disease (IBD), which comprises primarily Crohn’s disease (CD) and ulcerative colitis (UC), is characterized by chronic inflammation of the gastrointestinal tract, which causes debilitating symptoms such as abdominal pain, diarrhea, and weight loss. The causes of IBD are multifactorial and comprise interactions among genetic factors, environmental factors, the immune system, and microbiota (Abraham & Medzhitov, 2011; Ananthakrishnan, 2015; da Rosa Utiyama et al., 2001). The exact cause of IBD remains unclear, but immune dysregulation plays a pivotal role in its pathophysiologic processes. Recent studies have identified antinuclear antibodies (ANAs) as biomarkers significantly associated with autoimmune conditions, including IBD, which may reflect an aberrant immune response in affected individuals. The presence of ANAs may indicate heightened autoimmune activity and can complicate the clinical presentation of IBD. Several studies have reported that ANA positivity is more frequently observed in patients with UC than in those with CD (Folwaczny et al., 1997; García et al., 2022; Zauli et al., 1985). In patients with IBD, ANA positivity has also been observed both as part of the disease itself and in relation to therapy, particularly anti-tumor necrosis factor (anti-TNF) treatment, which is associated with ANA positivity in 20–45% of cases and the development of lupus-like syndrome (LLS) in up to 5% (Vaglio et al., 2018). However, data on the global prevalence of ANA in IBD, and on their associations with disease severity, treatment response, and seroconversion following biologic therapy, remain scarce (García et al., 2022).

In Taiwan, the incidence and prevalence of IBD are rising (Huang et al., 2025; Yen et al., 2024). The genetic susceptibility loci identified in Western patients, such as NOD2, occur less frequently in Taiwanese populations (Hsiao et al., 2007), whereas other loci may play more prominent roles (Tung et al., 2014). In Taiwan, traditional diets rich in rice, legumes, soy products, and tea appear protective against IBD, whereas Western diets high in red meat, dairy, and ultra-processed foods have been linked to increased disease risk, underscoring cultural differences that may partly explain regional variations in IBD prevalence (Meng et al., 2025). These distinct genetic and environmental factors may contribute to differences in the prevalence of both IBD and ANA positivity between Western and Asian populations (Guo et al., 2014).

The correlation between ANA positivity and the presence of extraintestinal manifestations (EIMs), disease severity, and response to biologic therapy has been studied (García et al., 2022); however, data specific to Asian populations remain limited. To address this knowledge gap, we examined the prevalence and risk factors of ANA in a Taiwanese cohort of patients with IBD.

## Materials and methods

### Patient population

This study was conducted at Changhua Christian Hospital, Changhua, Taiwan, from January 2017 to December 2024. We enrolled patients with diagnoses of IBD, including CD and UC, for whom ANA status was checked at initial diagnosis. Demographic, clinical, and laboratory data were reviewed retrospectively. Specific demographic and clinical parameters, such as disease activity, gastrointestinal tract involvement, EIMs, advanced therapy use, and treatment response, were evaluated.

### Method of ANA analysis

To measure ANA levels, we used HEp-20-10 cells (EUROIMMUN, Luebeck, Germany) in indirect immunofluorescence assay. Patients were classified as ANA positive if the titer values were ≥1:160 (García et al., 2022).

### Ethical considerations

The Ethics Committee of Changhua Christian Hospital approved the study protocol (CCH IRB No.: 250114). The requirement for informed consent was waived because of the retrospective nature of the study.

### Statistical analysis

Data for categorical variables were calculated as numbers and percentages. Those for continuous variables were calculated as means and standard deviations for normally distributed data and as medians and interquartile ranges (IQRs) for nonnormally distributed data. The distribution of continuous variables was examined in a one-sample Kolmogorov–Smirnov test. To compare categorical variables, we used the chi-square or Fisher’s exact test; to compare continuous variables, we used Student’s t test or the Mann–Whitney U-test, as appropriate. Multivariable logistic regression analysis was performed to identify factors associated with ANA positivity. We used MedCalc^®^ statistical software, version 23.0.2 (MedCalc Software Ltd, Ostend, Belgium; https://www.medcalc.org; 2024), to perform statistical analysis. Results with a *p* value of <0.05 was considered statistically significant.

## Results

### Clinical features of the study population

A total of 166 adult patients with IBD–57 with CD and 109 with UC–participated in this study. The baseline characteristics, with comparisons between CD and UC, are listed in Table 1. Joint EIMs, the most prevalent EIMs in the study population, affected similar proportions of patients with CD and UC (*p* = 0.93). Patients with CD, in comparison with those with UC, had higher rates of bowel resection (*p* < 0.01) and appendectomy (*p* < 0.05) and received more steroids (*p* < 0.05), azathioprine (*p* < 0.01), and biologics (*p* < 0.01). Similar biologic medications were used by patients with CD and UC; vedolizumab was used more commonly by patients with UC than by those with CD (*p* < 0.01). Of the patients with CD, the majority received one line of biologics, less than one third received a second line, and a tiny minority received a third line; of those with UC, the majority received one line of biologics, and fewer received second and third lines. ANA positivity was much more common among patients with UC than among those with CD (*p* < 0.05). In addition, CD was associated with certain environmental factors, such as smoking (*p* = 0.06), prior bowel resection (*p* < 0.01), and appendectomy (*p* < 0.05).

**Table 1:** The baseline characteristics of patients with IBD.

Variables	CD (*n* = 57)	UC (*n* = 109)	*p* value
Median age at diagnosis (IQR), years	30 (20.75–53.5)	37 (28.75–48.5)	0.18
Gender: male, *n*	36 (63.2%)	68 (62.4%)	0.92
Smoking, *n*			0.06
Current	3 (5.3%)	2 (1.8%)	
Ever	2 (3.5%)	0 (0%)	
No	52 (91.2%)	107 (98.2%)	
Alcohol consumption, *n* (%)	2 (3.5%)	2 (1.8%)	0.51
CD location, n*			–
L1	23 (40.4%)	–	
L2	4 (7%)	–	
L3	26 (45.6%)	–	
L4	4 (7%)	–	
CD behavior, *n*^†^			–
B1	21 (36.8%)	–	
B2	22 (38.6%)	–	
B3	14 (24.6%)	–	
Perianal disease	8 (14%)	–	–
UC location, *n*^‡^			–
E1	–	20 (18.3%)	
E2	–	33 (30.3%)	
E3	–	56 (51.4%)	
EIMs in joints, *n*	6 (10.5%)	11 (10.1%)	0.93
EIMs in skin, *n*	2 (3.5%)	4 (3.7%)	0.96
EIMs in eyes, *n*	0 (0%)	0 (0%)	–
ANA positivity, *n*	4 (7%)	22 (20.2%)	0.03
Bowel resection, *n*	15 (26.3%)	0 (0%)	<0.01
Appendectomy, *n*	2 (3.5%)	0 (0%)	<0.05
Use of Steroids, *n*	20 (35.1%)	20 (18.3%)	0.02
Use of 5-ASA, *n*	27 (47.4%)	72 (66.1%)	0.02
Use of AZA, *n*	23 (40.4%)	12 (11.0%)	<0.01
Use of any biologics, *n*	38 (66.7%)	32 (29.4%)	<0.01
Numbers of lines of biologics used, *n*			0.50
One	25 (65.8%)	21 (65.6%)	
Two	12 (31.6%)	7 (21.9%)	
Three	1 (2.6%)	4 (12.5%)	
Use of IFX, *n*^§^	7 (18.4%)	5 (15.6%)	0.76
Use of adalimumab (Humira), *n*^§^	16 (42.1%)	12 (37.5%)	0.70
Use of vedolizumab, *n*^§^	16 (42.1%)	24 (75.0%)	<0.01
Use of ustekinomab (Stelara), *n*^§^	13 (34.2%)	6 (18.8%)	0.15

**Notes:**

* L1, terminal ileum; L2, colon; L3, ileocolon; L4, multiple upper gastrointestinal locations.

† B1, nonstricturing and nonpenetrating inflammation; B2, stricturing inflammation; B3, penetrating inflammation.

‡ E1, proctitis; E2, left-sided colitis; E3, pancolitis.

§ Proportions were calculated as the number of patients receiving a particular biologic divided by the number receiving any biologic.

ANA, antinuclear antibody; 5-ASA, 5-aminosalicylic acid; AZA, azathioprine; CD, Crohn’s disease; EIM, extraintestinal manifestation; IFX, infliximab; IQR, interquartile range; UC, ulcerative colitis.

### The clinical correlation of specific IBD with ANA positivity

In this study, a total of 26 patients (15.7%) tested positive for ANA. The baseline characteristics, with comparisons between CD and UC, are listed in Table 2. ANA-positive patients were generally older at diagnosis than were ANA-negative patients (*p* < 0.05). We found no statistically significant differences in EIMs, surgical history, modality of medical therapy, or treatment with biologics between the two groups. Multivariate analysis identified age at diagnosis as a significant risk factor for ANA positivity (odds ratio (OR) 1.03, 95% confidence interval (CI) [1.00–1.06], *p* < 0.05). In contrast, Crohn’s disease was inversely associated with ANA positivity (OR 0.27, 95% CI [0.08–0.90], *p* < 0.05), suggesting a lower likelihood of ANA detection in CD compared with UC. Neither the use of biologics (OR 1.30, 95% CI [0.49–3.44], *p* = 0.60) nor the presence of extraintestinal manifestations (OR 2.30, 95% CI [0.76–6.96], *p* = 0.14) was significantly associated with ANA status (Table 3).

**Table 2:** Comparison of ANA-positive and ANA-negative patients with IBD.

Variables	ANA-positive (*n* = 26)	ANA-negative (*n* = 140)	*p* value
Median age at diagnosis (IQR), years	43 (32.0–53.0)	35 (25.0–48.0)	<0.05
Gender: male, *n*	14 (53.8%)	90 (64.3%)	0.31
Disease: CD, *n*	4 (15.4%)	53 (37.9%)	0.03
Any EIM, *n*	6 (23.1%)	17 (12.1%)	0.14
EIMs of joints, *n*	3 (11.5%)	14 (10.0%)	0.81
EIMs of skin, *n*	1 (3.8%)	5 (3.6%)	0.95
EIMs of eyes, *n*	0 (0%)	0 (0%)	–
Surgical history			
Bowel resection, *n*	0 (0%)	15 (10.7%)	0.08
Appendectomy, *n*	0 (0%)	2 (1.4%)	0.54
Medical therapy			
Use of steroids, *n*	6 (23.1%)	34 (24.3%)	0.90
Use of 5-ASA, *n*	20 (76.9%)	79 (56.4%)	0.05
Use of AZA, *n*	5 (19.2%)	30 (21.4%)	0.80
Use of any biologics, *n*	10 (38.5%)	60 (42.9%)	0.66
Number of lines of biologics used, *n*			0.53
One	7 (70%)	39 (65%)	
Two	3 (30%)	16 (26.7%)	
Three	0 (0%)	5 (8.3%)	
CD location, n*			0.47
L1	1 (25.0%)	22 (41.5%)	
L2	1 (25.0%)	3 (5.7%)	
L3	1 (50.0%)	24 (45.3%)	
L4	0 (0%)	4 (7.5%)	
CD behavior, *n*^†^			0.03
B1	4 (100%)	17 (32.1%)	
B2	0 (0%)	22 (41.5%)	
B3	0 (0%)	14 (26.4%)	
UC location, *n*^‡^			0.11
E1	1 (4.5%)	19 (21.8%)	
E2	6 (27.3%)	27 (31.0%)	
E3	15 (68.2%)	41 (47.1%)	

**Notes:**

* L1, terminal ileum; L2, colon; L3, ileocolon; L4, multiple upper gastrointestinal locations.

† B1, nonstricturing and nonpenetrating inflammation; B2, stricturing inflammation; B3, penetrating inflammation.

‡ E1, proctitis; E2, left-sided colitis; E3, pancolitis.

ANA, antinuclear antibody; CD, Crohn’s disease; EIM, extraintestinal manifestation; IBD, inflammatory bowel disease; IQR, interquartile range; UC, ulcerative colitis.

**Table 3:** Risk factors related to ANA positivity.

Variables	*p* value	OR	95% CI
Age at diagnosis	<0.05	1.03	[1.00–1.06]
Disease type: CD	<0.05	0.27	[0.08–0.90]
Use of biologics	0.60	1.30	[0.45–3.44]
Presence of EIM	0.14	2.30	[0.76–6.96]

**Note:**

ANA, antinuclear antibody; CD, Crohn’s disease; CI, confidence interval; EIM, extraintestinal manifestation; OR, odds ratio.

When evaluating response to first-line advanced therapy, no significant association was observed between baseline ANA status and treatment outcomes. Among patients receiving anti-TNF therapy, 80.8% of ANA-negative patients and 100% of ANA-positive patients achieved a primary response (*p* = 0.34). Similarly, in those treated with anti-integrin therapy, 92.0% of ANA-negative and 100% of ANA-positive patients responded (*p* = 0.48) (Table 4).

**Table 4:** Response to first-line advanced therapy according to ANA status at baseline.

Anti-TNF as first-line medication (*n* = 30; *p* = 0.34)		
Primary response	ANA-negative patients (*n* = 26 [86.7%])	ANA-positive patients (*n* = 4 [13.3%])
No response	5 (19.2%)	0 (0%)
Response	21 (80.8%)	4 (100%)
Anti-integrin as first-line medication (*n* = 31; *p* = 0.48)		
Primary response	ANA-negative patients (*n* = 25 [80.6%])	ANA-positive patients (*n* = 6 [19.4%])
No response	2 (8.0%)	0 (0%)
Response	23 (92.0%)	6 (100%)

**Note:**

ANA, antinuclear antibody; TNF, tumor necrosis factor.

### ANA pattern frequency and titers

Among the 26 ANA-positive patients (22 UC patients and four CD patients), the ANA patterns and titers were further classified into various anti-cell (AC) categories, according to the International Consensus on Antinuclear Antibody Patterns (ICAP) (Chan et al., 2015) coding of HEp-2 patterns on immunofluorescence assay (Tables 5 and 6). Of the 26 patients, 6 exhibited two AC patterns, and two exhibited three AC patterns. When stratifying patients into UC and CD groups, patients with UC demonstrated a higher frequency and broader diversity of ANA patterns. The most common pattern was AC1 (*n* = 14), followed by AC4 (*n* = 4), AC3 (*n* = 3), and AC8 (*n* = 3). Less frequent patterns included AC2, AC19, AC21, AC26, AC27, and AC6 (all *n* = 1–2). ANA titers in UC ranged widely from 1:160 to 1:2,560, with AC3 showing particularly high titers (median 1:640) compared with other subtypes. In contrast, Crohn’s disease (CD) showed fewer ANA-positive cases and more heterogeneous patterns. AC1 was identified in two patients (titers 1:320–1:640), while single cases of AC3 (1:160), AC11 (1:2,560), and AC21 (1:2,560) were observed.

**Table 5:** ANA patterns: frequency and titers in UC patients*.

Anti-cell (AC) pattern	ANA titers, *n*					*n* (%)
	1:160	1:320	1:640	1:1,280	1:2,560	
AC1	7	3	0	3	1	14 (45.16%)
AC2	1	1	0	0	0	2 (6.45%)
AC3	1	0	1	1	0	3 (9.68%)
AC4	1	3	0	0	0	4 (12.90%)
AC6	0	1	0	0	0	1 (3.23%)
AC8	2	1	0	0	0	3 (9.68%)
AC19	1	0	0	0	0	1 (3.23%)
AC21	1	0	0	0	1	1 (3.23%)
AC26	0	1	0	0	0	1 (3.23%)
AC27	1	0	0	0	0	1 (3.23%)
*n* (%)	15 (48.39%)	10 (32.26%)	1 (3.23%)	4 (12.90%)	1 (3.23%)	31 (100%)

**Notes:**

* Of the 22 ANA-positive patients, 5 had two AC patterns and 2 had three AC patterns.

ANA, antinuclear antibody.

**Table 6:** ANA patterns: frequency and titers in CD patients*.

Anti-cell (AC) pattern	ANA titers, *n*					*n* (%)
	1:160	1:320	1:640	1:1,280	1:2,560	
AC1	0	1	1	0	0	2 (40%)
AC3	0	0	0	0	1	1 (20%)
AC11	1	0	0	0	0	1 (20%)
AC21	0	0	0	0	1	1 (20%)
*n* (%)	1 (20%)	1 (20%)	1 (20%)	0 (0%)	2 (40%)	5 (100%)

**Notes:**

* Of the 4 ANA positive patients, 1 had two AC pattern.

ANA, antinuclear antibody.

## Discussion

To our knowledge, our study is one of the first studies on the prevalence and risk factors of ANA positivity among patients with IBD in Taiwan and investigation of the relationship between the presence of ANA before any treatment and the types of and response to medical therapy. We have collected data on the frequency of ANA patterns and titers in our patient population.

Our findings indicated that ANA positivity had a prevalence of 16.25% among patients with IBD. Previous studies had also shown that the prevalence of ANA positivity among patients with IBD (13.6–53.5%) was higher than that in the global healthy population (Barahona-Garrido et al., 2009; Garcia-Planella et al., 2003; García et al., 2022); however, recent studies indicate that the prevalence of ANA titers of >1:80 is increasing in healthy populations, ranging from 14.01% to 16.1% (Dinse et al., 2022; Li et al., 2019). As ANA prevalence increases in the healthy population, clinicians must be more cautious and rigorous when interpreting the relationship between ANA positivity and autoimmunity. According to our findings, ANA positivity was not associated with gender, but it was more common among older individuals and among patients with UC. In the past, many investigators have reported a higher proportion of ANA positivity among patients with UC than among those with CD (Folwaczny et al., 1997; García et al., 2022; Zauli et al., 1985); however, the underlying pathophysiologic reasons remain unclear. IBD is a complex, immunologically mediated disease involving interactions among genetic, immunologic, and environmental factors and microbiota; therefore, the higher prevalence of ANA positivity in UC may reflect the autoimmune nature of the disease, whereas CD may be more genetically determined, environmentally related, or associated with autoinflammation. Our study also identified certain environmental factors, such as smoking, prior bowel resection, and appendectomy that were correlated with CD.

ANA positivity was not predictive of EIM presence in our study (Table 2). Previous studies also showed little association between ANA positivity and EIMs (Folwaczny et al., 1997; García et al., 2022). The exact relationship between IBD and EIMs remains unclear. Hypotheses regarding the pathophysiologic process of the extensive immune response include ectopic expression of adhesion molecules and chemokines outside the gut, microbial antigen cross-reactivity, microbial antigen translocation, a shift in inflammatory tone, or systemic changes in innate immune function (Faggiani et al., 2024). High titers of ANA represent high sensitivity but low specificity for immune function; thus, ANA data provide valuable clinical clues for the diagnosis of autoimmune diseases and autoimmune reactions. The negative association between ANA and EIMs may suggest that autoimmunity is less associated with EIM in our IBD patiens.

In our cohort, we found no correlation between initial ANA positivity before any treatment and types of therapy (steroids, azathioprine, or any biologics). According to the official recommendations of the European Crohn’s and Colitis Organization and the American Gastroenterological Association (Feuerstein et al., 2020; Gordon et al., 2024; Raine et al., 2022), the choice of medical therapy was based on disease severity, response to clinical treatment, and the physician’s clinical judgment. For most autoimmune diseases, ANA, representing immune titers for the diagnosis of autoimmune disease, is not considered in the determination of disease severity. In IBD, as with other autoimmune diseases, ANA probably plays an insignificant role in disease severity and is therefore unrelated to the type of medical therapy. Furthermore, patients with IBD and ANA positivity responded similar to both anti-TNF medication (*p* = 0.34) and anti-integrin (*p* = 0.48) in our study than ANA negative population. The ANA positivity was not correlated with the use of second- or third-line biologics (*p* = 0.53). In contrast, ANA was previously reported to be linked to poor response to anti-TNF therapy; the poor responses included the development of adverse events (Santos-Antunes et al., 2016), infusion reactions, and shorter duration of treatment response (Baert et al., 2003; Theodoraki et al., 2022). Although our study had a small sample size bias, a possible explanation for the inconsistency could be the different population characteristics, such as ethnicity and environment. The population in our study consisted entirely of Asian individuals in Taiwan; Asian ethnicity is regarded as a “low-incidence” ethnicity and Taiwan as a “low-incidence” region in the world. The genetic and environmental factors may not only reflect the different incidences of IBD but also contribute to disease severity and response to biologic therapy.

In contrast to previous studies, we not only measured ANA titers in patients with IBD but also investigated the ANA patterns according to the ICAP classifications within the population. To our knowledge, no studies to date have specifically reported ANA patterns in IBD using the ICAP classification (García et al., 2022). In our cohort, the most common ANA titer was 1:160, and the most common ANA pattern was AC1, followed by AC3 and AC4. All three ANA patterns reflect the nucleolar pattern of ANA staining. The AC1 pattern is most common in patients with systemic lupus erythematosus and type 1 autoimmune hepatitis, whereas the AC3 and AC4 patterns are associated with systemic sclerosis (scleroderma). However, because of the relatively small sample size, no definitive clinical implications can be drawn from the indirect immunofluorescence findings associated with ANA positivity in our cohort. Nonetheless, our observation of AC-1, AC-3, and AC-4 as the predominant ANA patterns in IBD may indicate a partial overlap with systemic autoimmunity, but without the same clinical significance. In systemic autoimmune diseases, these patterns often serve as important diagnostic or prognostic markers (Chan et al., 2015; Damoiseaux et al., 2019) . In contrast, their presence in IBD most likely reflects broader immune dysregulation rather than a disease-specific signature. Thus, further larger-scale studies are required to explore the different ICAP patterns in the IBD population.

Our investigation had several limitations. First, the number of cases was relatively low because it was conducted in a single medical center and because the prevalence of IBD among the Taiwanese population is low (Chung et al., 2025; Lee, Yen & Chen, 2025; Yang et al., 2022, 2024; Yen et al., 2023). Second, the study was retrospective, and ANA titers were not periodically monitored to observe their chronologic changes. Because ANA titers were measured at baseline, before initiation of any biologic or immunomodulator therapy, the observed ANA positivity in this Taiwanese IBD cohort likely reflects pre-existing autoimmunity rather than drug-induced autoantibody formation and thus occurred independently of TNFi exposure. In patients with undetectable ANA levels before the beginning of biologic therapy, earlier research demonstrated positive seroconversion of ANA in follow-up visits during anti-TNF therapy, which may lead to the treatment failure of anti-TNF medications (Atzeni et al., 2005; Atzeni & Sarzi-Puttini, 2008; García et al., 2022; Hanauer, 1999; Theodoraki et al., 2022; Vermeire et al., 2003). Sequential reduction of ANA titers after immunomodulator therapy and during simultaneous administration of an immunomodulator and biologic therapy was reported previously (Beigel et al., 2011; García et al., 2022). We did not assess sequential changes in ANA titers with respect to age, disease severity, treatment modality particularly newer agents such as interleukin inhibitors (Chung et al., 2025) or Janus kinase (JAK) inhibitors (Chen, Yen & Chen, 2025) and other potential influencing factors in the present study. Third, subsequent autoantibody workups were inconsistent because of referrals to various rheumatologists. When a patient tests positive for ANA, a rheumatologic evaluation is recommended, but further investigation may depend on individual rheumatologists’ practice. Moreover, antineutrophil cytoplasmic antibody (ANCA) or other autoimmune profiles were not checked because ANA is only routinely screened at the baseline during the initial diagnosis of IBD. Except for ANA, other autoantibodies, such as ANCA, anti-CBir1, and anti–Saccaromyces cerevisiae antibody (ASCA) (Vermeire et al., 2008), are also associated with IBD. Last, we did not exclude patients with other autoimmune diseases, such as systemic lupus erythematosus, Sjögren’s syndrome, and autoimmune hepatitis, from our study. Such patients may have been ANA positive as a result of underlying autoimmune disease, which would have led to statistical bias in our study.

## Conclusions

This study revealed that although ANA positivity is relatively common among Taiwanese patients with IBD, particularly older individuals and those with UC. To our knowledge, this study is the first investigation of the relationship between the presence of ANA before any treatment and the modality of medical therapy. Nevertheless, several important questions remain. Future studies should determine whether ANA positivity predicts long-term outcomes such as treatment durability, risk of adverse events, or development of other autoimmune comorbidities. In addition, prospective studies with larger multi-center Asian cohorts and longitudinal monitoring of ANA titers are needed to clarify whether ANA seroconversion during biologic therapy influences treatment response. Exploration of other autoantibodies in parallel with ANA may further refine our understanding of autoimmune profiles in Asian patients with IBD.

## Supplemental Information

10.7717/peerj.20474/supp-1 Supplemental Information 1 STROBE Checklist.

10.7717/peerj.20474/supp-2 Supplemental Information 2 Data.

## Additional Information and Declarations

### Data Availability

The following information was supplied regarding data availability:

The raw data is available in the Supplemental Files.
